# Time-Frequency Analysis of Cardiovascular and Cardiorespiratory Interactions During Orthostatic Stress by Extended Partial Directed Coherence

**DOI:** 10.3390/e21050468

**Published:** 2019-05-05

**Authors:** Sonia Charleston-Villalobos, Sina Reulecke, Andreas Voss, Mahmood R. Azimi-Sadjadi, Ramón González-Camarena, Mercedes J. Gaitán-González, Jesús A. González-Hermosillo, Guadalupe Hernández-Pacheco, Steffen Schulz, Tomás Aljama-Corrales

**Affiliations:** 1 Department of Electrical Engineering, Universidad Autónoma Metropolitana, Mexico City 09340, Mexico; 2 Institute of Innovative Health Technologies, Ernst-Abbe-Hochschule Jena, 07745 Jena, Germany; 3 Department of Electrical and Computer Engineering, Colorado State University, Fort Collins, CO 80523, USA; 4 Department of Health Science, Universidad Autónoma Metropolitana, Mexico City 09340, Mexico; 5 National Institute of Cardiology, Mexico City 14080, Mexico

**Keywords:** time-frequency representation, extended partial directed coherence, cardiovascular interactions, cardiorespiratory interactions, orthostatic intolerance, gender effects

## Abstract

In this study, the linear method of extended partial directed coherence (ePDC) was applied to establish the temporal dynamic behavior of cardiovascular and cardiorespiratory interactions during orthostatic stress at a 70° head-up tilt (HUT) test on young age-matched healthy subjects and patients with orthostatic intolerance (OI), both male and female. Twenty 5-min windows were used to analyze the minute-wise progression of interactions from 5 min in a supine position (baseline, BL) until 18 min of the orthostatic phase (OP) without including pre-syncopal phases. Gender differences in controls were present in cardiorespiratory interactions during OP without compromised autonomic regulation. However in patients, analysis by ePDC revealed considerable dynamic alterations within cardiovascular and cardiorespiratory interactions over the temporal course during the HUT test. Considering the young female patients with OI, the information flow from heart rate to systolic blood pressure (mechanical modulation) was already increased before the tilt-up, the information flow from systolic blood pressure to heart rate (neural baroreflex) increased during OP, while the information flow from respiration to heart rate (respiratory sinus arrhythmia) decreased during the complete HUT test. Findings revealed impaired cardiovascular interactions in patients with orthostatic intolerance and confirmed the usefulness of ePDC for causality analysis.

## 1. Introduction

During recent years, the investigation of causality or driver-response relationships between physiological variables has become of great interest among researchers. The analysis of multivariate interactions might provide more insights into abnormalities or impairments due to cardiovascular or cerebral pathologies in addition to univariate variability or coupling analyses. Biological signals are rich of oscillatory content which gave rise to the development of linear methods that are strictly related to the frequency domain representation of multichannel data [1]. The linear frequency domain framework enabled the estimation of causality between specific oscillatory components within cardiovascular and respiratory signals. Physiological time series can be represented by a multivariate autoregressive (MVAR) model which allows deriving frequency domain measures of causality based on the model coefficients. One of the most used measures to quantify direct causality as a function of frequency is the partial directed coherence (PDC) and its refined versions [2]. The PDC is derived from a factorization of the partial coherence able to elicit causality from the modeled interactions. The traditional PDC was defined for a strictly causal MVAR model including only lagged effects. Recently, the extended PDC (ePDC) using an extended MVAR (eMVAR) model has been proposed to account for both instantaneous and lagged effects [3]. A meaningful rationale for using ePDC is that cardiovascular and cardiorespiratory interactions can be instantaneous (zero beat delay) [4]. However, the ePDC requires a prior definition of possible instantaneous effects which can be realized by predefining the temporal occurrence of physiological events if present. Furthermore, the proposed ePDC should be applied to stationary time series. In practical analysis, local stationarity can be assumed by applying short-term shifted windows to the available physiological data. Linear time-invariant analyses like the eMVAR-based approach remain of great appeal for the study of physiological interactions. Therefore, a linear approximation might be a reasonable first approach to describe causality between physiological processes.

Cardiovascular variables such as heart rate (HR) and blood pressure (BP) are characterized by respiratory related fluctuations. Blood pressure decreases during inspiration and increases during expiration due to mechanical modulation [5]. Respiration and HR are further coupled, and it is known as the respiratory sinus arrhythmia (RSA) [6]. Hereby, decreased vagal activity during inspiration causes HR accelerations, while increased vagal activity during expiration leads to HR decelerations [7]. Cardiorespiratory coupling is dependent on respiratory frequency [8], in which increased respiratory frequency, e.g., during stress, is supposed to progressively reduce RSA [9]. Cardiopulmonary interactions from cardiovascular variables to respiration are less pronounced and are still under investigation [10]. Besides the interactions on HR and BP mediated by respiration, interactions within the cardiovascular system exist serving the regulation of BP [7,11,12]. On one side, HR affects BP via mechanical feed-forward modulations, where HR accelerations/decelerations induce increases/decreases in BP. On the other side, BP influences HR based on fast feedback mechanisms via the neural baroreflex. The baroreflex is one of the most important neural mechanisms in the cardiovascular control, and is responsible for regulating blood pressure and ensuring adequate perfusion [13] as well as reestablishing BP adequately during an orthostatic challenge [14]. Consequently, four interactions can be established based on cardiovascular and respiratory physiology, i.e., from respiration to both HR and BP in addition to bidirectional interactions between HR and BP.

Signal processing techniques in the time, frequency and information domains [11] have been applied to study interactions during an orthostatic challenge, including Granger causality [15], transfer entropy [11,16], measures of synchronization [17] as well as partial directed coherence [18]. In agreement with the sympathetic activation accompanied by vagal withdrawal caused by orthostatic stress, interactions from BP to HR increased the reflecting augmented baroreflex function, interactions from HR to BP did not change, interactions from respiration to HR decreased corresponding to dampened RSA and interactions from respiration to BP were unchanged or increased in orthostatic stress [11,12,15,19,20,21]. In previous applications of ePDC to cardiovascular data from healthy subjects, ePDC increased in the low frequency band from BP to HR concurrent with the tilt-induced sympathetic activation [18,22]. In addition, ePDC in the high frequency band confirmed unidirectional interactions from respiration to both BP and HR without differences between supine and tilt position. Concerning autonomic disorders such as orthostatic intolerance (OI), reduced interactions from BP to HR due to decreased baroreflex sensitivity were observed after a prolonged orthostatic phase or just before the occurrence of pre-syncope as one feature of cardiovascular dysregulation [4,11,12,20,23].

Previous studies with ePDC are theoretically well-established with simulations, but their scope of applications to cardiovascular data has been limited. Moreover, the temporal course of interactions in healthy and sick populations facing a stressor has not yet been obtained. In particular, the temporal dynamics of interactions during the beginning of the orthostatic phase have not yet been studied when considering healthy subjects and patients susceptible to orthostatic intolerance before the occurrence of pre-syncope, with the additional factor to account for gender effects in both cohorts. Therefore, the objective of the present study was to investigate the time course of the dynamic behavior of cardiovascular and cardiorespiratory interactions applying ePDC to the segmented multivariate process, leading to a time-frequency representation of interactions. There are different methods to compute a time-frequency representation of the time series of variability based on instantaneous amplitudes and frequencies, but no information flow between processes is included, as with ePDC. Furthermore, in the time-frequency scheme by ePDC, the dependence of the process *y_i_* on *y_j_* is computed without considering the influence of a third process. We hypothesized that temporal dynamic analyses during an orthostatic challenge by ePDC will provide evidence for gender differences, as well as for alterations within cardiovascular and cardiorespiratory interactions in patients that might help to provide more insights into the autonomic disorder. The present paper may contribute to bringing new readers to an interaction analysis during orthostatic stress, among other effects.

Since PDC is a directional quantity, it can be related to the concept of “directed” connectivity, and then to the direction of information flow. In fact, there are some studies that have focused on confirming the relationship between PDC and information flow and, consequently, establishing a unified framework for causal inference that links information-theoretic methods as transfer entropy, and PDC based on autoregressive models [24,25]. The advantage of PDC is that in using the autoregressive approach, a straightforward decomposition by frequency is possible, in other words, causality is estimated within the well-understood framework of MVAR modeling. Consequently, in the present paper the term information flow is used to describe the directed connectivity in the cardiovascular and respiratory network.

## 2. Materials and Methods 

### 2.1. Subjects and Experimental Protocol

In the present study, a passive head-up tilt (HUT) test was performed in a total of 53 adult subjects divided into four age-matched groups including 13 male and 13 female controls as well as 6 male and 21 female patients diagnosed with orthostatic intolerance, as shown in Table 1. None of the healthy subjects had clinical signs of autonomic disorders or were receiving any medication. All subjects were breathing spontaneously during the protocol. High-resolution electrocardiogram (ECG, 1000 Hz sampling frequency), continuous non-invasive arterial pressure (CNAP, 100 Hz sampling frequency) and respiration (10 Hz sampling frequency) were simultaneously recorded using a Task Force Monitor 3040i (CNSystems, Graz, Austria). The data recording began with a rest period of 5 min in a supine position, followed by an orthostatic phase of 30 min at 70°. All controls completed the HUT test without any problems. During the upright position, patients demonstrated at least one of the symptoms of orthostatic intolerance, including dizziness, headache, nausea, hyperventilation, heat, shiver, weakness and anxiety. If a patient showed pre-syncopal symptoms, the HUT test was terminated by returning the tilt table back to the supine position. None of the patients demonstrated symptoms of pre-syncope until 18 min of the orthostatic phase. The protocol was performed during the morning in a laboratory with controlled environment, under informed consent according to the Declaration of Helsinki, and approved by the Ethics Committee of the National Institute of Cardiology at Mexico City.

### 2.2. Pre-Processing and Data Analysis

A time series of successive beat-to-beat intervals (BBI), respiratory amplitude (RESP) at BBI onset as well as systolic (SYS) blood pressure were extracted from the recorded ECG, respiration and CNAP signals. The value of SYS (*n*) was taken within the current BBI (*n*). All extracted time series were manually reviewed and corrected. For ePDC analysis, the time series were resampled at 2 Hz using spline interpolation and normalized to zero mean and unit variance. With reference to the Introduction section, a total of four directional bivariate interactions were analyzed including SYS→BBI, BBI→SYS, RESP→BBI and RESP→SYS. Dynamic data analysis was performed by applying 5-min windows with a shift of 1 min to investigate the temporal dynamic behavior of causal interactions during an orthostatic challenge. A total of 20 windows (until 18 min of orthostatic phase (OP)) were extracted, corresponding to the following stages: baseline (BL, supine position): window 1; transition (T, including tilt-up to OP): windows 2–6 and orthostatic phase (OP): windows 7–20. The ePDC parameters were calculated for all windows and used to evaluate statistical differences between groups. The effect of gender was investigated in controls (FemCon vs. MaleCon) and in patients (FemPat vs. MalePat). In addition, the effect of orthostatic intolerance was analyzed in women (FemCon vs. FemPat) as well as in men (MaleCon vs. MalePat).

### 2.3. Cardiovascular and Cardiorespiratory Interactions by ePDC

#### 2.3.1. Time-Frequency Analysis of Interactions

A time-frequency representation (TFR) framework for studying the temporal evolution of interactions of cardiovascular and cardiorespiratory systems can be formulated in terms of ePDC applied on segmented multivariate processes. TFR can be derived using a segmented eMVAR model whose general expression is as follows:

(1) $$Y\left(n,w_{l}\right)=\overset{q}{\underset{k=0}{\sum }}B\left(k,w_{l}\right)Y\left(n-k,w_{l}\right)+U\left(n,w_{l}\right),$$

where $Y\left(n,w_{l}\right)={\left[y_{1}\left(n,w_{l}\right),\ldots ,y_{M}\left(n,w_{l}\right)\right]}^{T}$ is a set of M simultaneously observed zero-mean time series obtained by sampling the stochastic process Y at the time instant n within the time window $w_{l}$, l=1,…,L. Also, $B\left(k,w_{l}\right)$ are M×M coefficient matrices in which the element $b_{ij}\left(k,w_{l}\right)$ describes the dependence of $y_{i}\left(n,w_{l}\right)$ on $y_{j}\left(n-k,w_{l}\right)\left(i,j=1,\ldots ,M;k=0,1,\ldots ,q;l=1,\ldots ,L\right),$ and $U\left(n,w_{l}\right)={\left[u_{1}\left(n,w_{l}\right),\ldots ,u_{M}\left(n,w_{l}\right)\right]}^{T}$ is the driving process formed by white and uncorrelated noise processes with covariance matrix $\Lambda \left(w_{l}\right)=\mathrm{cov}\left(U\left(n,w_{l}\right)\right)=\mathrm{diag}\left(\lambda_{i}^{2}\right).$ Therefore, the eMVAR model in Equation (1) allows us to derive definitions of causality in terms of the off-diagonal elements of the coefficient matrices $B\left(k,w_{l}\right)$. It is worthy to note that instantaneous effects from one scalar process to another are represented by the matrix $B\left(0,w_{l}\right).$ The definition of extended causality includes both instantaneous (*k* = 0) and lagged (*k* > 0) causal influences between time series.

A relevant issue for obtaining the eMVAR model is the estimation of the matrices $B\left(k,w_{l}\right),k=0,\ldots ,q$. To obtain these matrices, it is important to consider the relation between the strictly causal MVAR model and the extended one. First, the segmented eMVAR model in Equation (1) can be rewritten as:

(2) $$Y\left(n,w_{l}\right)=\overset{q}{\underset{k=1}{\sum }}{\left(I-B\left(0,w_{l}\right)\right)}^{-1}B\left(k,w_{l}\right)Y\left(n-k,w_{l}\right)+{\left(I-B\left(0,w_{l}\right)\right)}^{-1}U\left(n,w_{l}\right),$$

if the matrix $B\left(0,w_{l}\right)$ has all its entries equal to zero, a strictly causal MVAR model is obtained. Second, the general expression of a strictly causal MVAR model can be written as:

(3) $$Y\left(n,w_{l}\right)=\overset{q}{\underset{k=1}{\sum }}A\left(k,w_{l}\right)Y\left(n-k,w_{l}\right)+W_{C}\left(n,w_{l}\right),$$

where each element $a_{ij}\left(k,w_{l}\right)$ describes the dependence of $y_{i}\left(n,w_{l}\right)$ on $y_{j}\left(n-k,w_{l}\right),$ and $W_{c}\left(n,w_{l}\right)={\left[w_{c1}\left(n,w_{l}\right),\ldots ,w_{cM}\left(n,w_{l}\right)\right]}^{T}$ is the driving process. Consequently, comparing Equations (2) and (3), $A\left(k,w_{l}\right)={\left(I-B\left(0,w_{l}\right)\right)}^{-1}B\left(k,w_{l}\right)$ for k=1,…,q, and $W_{c}\left(n,w_{l}\right)={\left(I-B\left(0,w_{l}\right)\right)}^{-1}U\left(n,w_{l}\right).$ Also, the covariance matrix of the process $W_{C}$ is given by $\Sigma \left(w_{l}\right)=L\left(w_{l}\right)\Lambda \left(w_{l}\right)L^{T}\left(w_{l}\right),$ where $L\left(w_{l}\right)={\left(I-B\left(0,w_{l}\right)\right)}^{-1};$ if instantaneous effects are present, the matrix $\Sigma \left(w_{l}\right)$ is not diagonal. Then, from a strictly causal MVAR model and from a Cholesky decomposition of $\Sigma \left(w_{l}\right)$, getting the matrix $L\left(w_{l}\right)$ is possible and, consequently, the matrices of the eMVAR model $B\left(k,w_{l}\right)$ can be estimated as $B\left(k,w_{l}\right)=L{\left(w_{l}\right)}^{-1}A\left(k,w_{l}\right)$. It is important to note that $L\left(w_{l}\right)$ is a lower triangular matrix, implying that $B\left(0,w_{l}\right)$ is also a lower triangular matrix with a null diagonal. In practical applications, to achieve the former constraint, the analyzed multivariate time series need to be ordered for each j<i, meaning that instantaneous effects are allowed from $y_{j}\left(n,w_{l}\right)$ to $y_{i}\left(n,w_{l}\right)$, i.e., $b_{ij}\left(0,w_{l}\right)\neq 0$ but not the other way around [3,18]. In summary, the strictly causal MVAR model is estimated by classic regression methods, such as the standard least-squares identification, and from there the eMVAR model is obtained. In this work, the time series were segmented in 20 time windows, i.e., L = 20 and three time series were used, i.e., M = 3. The time series were ordered knowing that temporally during one heartbeat the first measure that occurs is the respiratory amplitude (RESP) at BBI onset, the second measure is the systolic blood pressure (SYS) and finally, the beat-to-beat interval (BBI) is determined at the end of the heartbeat. Therefore, instantaneous effects (within the same heartbeat) are only possible for the interactions RESP→SYS, RESP→BBI and SYS→BBI. Consequently, the order of the time series for the extended MVAR model was y_1_ = RESP, y_2_ = SYS and y_3_ = BBI. Furthermore, the model order *q* was determined using the minimum value of the Akaike figure of merit, defined as $\mathrm{AIC}\left(q,w_{l}\right)=N\log\left(\det\left(\Sigma \left(w_{l}\right)\right)\right)+2M^{2}q.$

Once the eMVAR model is estimated, the TFR of the cardiovascular and cardiorespiratory interactions can be obtained from the extended partial directed coherence (ePDC). Generally speaking, taking the Fourier transform of Equation (1), the eMVAR model in the frequency domain corresponds to $Y\left(f,w_{l}\right)=B\left(f,w_{l}\right)Y\left(f,w_{l}\right)+U\left(f,w_{l}\right)$, where $B\left(f,w_{l}\right)=\overset{q}{\underset{k=0}{\sum }}B\left(k,w_{l}\right)e^{-j2\pi fk}$. Also, considering the transfer function from $U\left(f,w_{l}\right)$ to $Y\left(f,w_{l}\right)$, the spectral representation may be rewritten as $Y\left(f,w_{l}\right)=G\left(f,w_{l}\right)U\left(f,w_{l}\right)$, where $G\left(f,w_{l}\right)={\left[I-B\left(f,w_{l}\right)\right]}^{-1}=\bar{B}{\left(f,w_{l}\right)}^{-1}$ is the M×M transfer matrix in the frequency domain. Theoretically, the partial coherence (PC) between the processes $y_{i}$ and $y_{j}$, i.e., $\Pi_{ij}\left(f,w_{l}\right)$ is defined as:

(4) $$\Pi_{ij}\left(f,w_{l}\right)=\frac{P_{ij}\left(f,w_{l}\right)}{\sqrt{P_{ii}\left(f,w_{l}\right)P_{jj}\left(f,w_{l}\right)}}$$

where $P_{YY}\left(f,w_{l}\right)=S_{YY}^{-1}\left(f,w_{l}\right),$ i.e., $P_{YY}\left(f,w_{l}\right)$ is the inverse of the spectral matrix of the MVAR process. From $Y\left(f,w_{l}\right)=G\left(f,w_{l}\right)U\left(f,w_{l}\right),$ it can be demonstrated that the spectral matrix $S_{YY}\left(f,w_{l}\right)=G\left(f,w_{l}\right)\Lambda \left(w_{l}\right)G^{H}\left(f,w_{l}\right)$ and $P_{YY}\left(f,w_{l}\right)={\bar{B}}^{H}\left(f,w_{l}\right)\Lambda^{-1}\left(w_{l}\right)\bar{B}\left(f,w_{l}\right).$ Consequently, the PC can be rewritten and factorized as follows:

(5) $$\Pi_{ij}\left(f,w_{l}\right)=\overset{M}{\underset{m=1}{\sum }}\frac{\frac{1}{\lambda_{m}}{\bar{B}}_{mj}\left(f,w_{l}\right)\frac{1}{\lambda_{m}}{\bar{B}}_{mi}^{*}\left(f,w_{l}\right)}{\sqrt{P_{ii}\left(f,w_{l}\right)}\sqrt{P_{jj}\left(f,w_{l}\right)}}=\overset{M}{\underset{m=1}{\sum }}\chi_{mj}\left(f,w_{l}\right)\chi_{mi}^{*}\left(f,w_{l}\right),$$

where the extended partial directed coherence (ePDC), $\chi_{ij}\left(f,w_{l}\right)$, is given by:

(6) $$\chi_{ij}\left(f,w_{l}\right)=\frac{\left({\frac{1}{\lambda }}_{i}\right){\bar{B}}_{ij}\left(f,w_{l}\right)}{\sqrt{\sum_{m=1}^{M}\left(1/\lambda_{m}^{2}\right){\left|{\bar{B}}_{mj}\left(f,w_{l}\right)\right|}^{2}}}$$

and $\lambda_{i}^{2}$ is the variance of the noise $u_{i}$ in Equation (1), with ${\bar{B}}_{ij}\left(f,w_{l}\right)=\delta_{ij}-\overset{q}{\underset{k=0}{\sum }}b_{ij}\left(k,w_{l}\right)e^{-j2\pi fk}.$

The squared modulus of ePDC, ${\left|\chi_{ij}\left(f,w_{l}\right)\right|}^{2}$, is a measure of direct causality, or information flow, as a function of frequency f and $w_{l}$ that estimates the influence of $y_{j}$ onto $y_{i}.$ The ePDC is an asymmetric causality measure which elicits directional information. The ePDC is normalized with respect to the structure that sends the signal, taking values between 0, the absence of causality, and 1, representing full causality between two processes. ePDC is the normalized proportion of $P_{jj}\left(f,w_{l}\right)$ of the input process $y_{j}$ which is sent to the output process $y_{i}$. The software Matlab R2013a by MathWorks was used for data analysis.

#### 2.3.2. Surrogate Data Analysis

The assessment of statistical significance of the derived causality measures is of great practical importance. Nonzero values are likely to occur at some frequencies, even in the case of absence of a true interaction between the two considered processes. This problem is commonly faced by means of statistical hypothesis testing procedures based on setting a threshold for significance at the upper limit of the confidence interval of the considered index. For short data segments, empirical distributions of the considered index can be obtained using a set of surrogate time series by preserving the power spectra, but destroying their coupling. The surrogates method based on Fourier Transform (FT) was applied, where causality is selectively destroyed only over the direction under study and preserved over the remaining directions. Once the eMVAR model is estimated, if there is a direct causality from the process $y_{j}$ to $y_{i},$ the corresponding coefficients $b_{ij}\left(k,w_{l}\right),k=0,\ldots ,q,$ are set to zero, and from there the surrogates are obtained, with details of the procedure being found in [26]. In the present study, based on previous experience, the threshold was estimated using N = 100 surrogates applied to each cardiovascular temporal window with length of 600 samples. A thresholding procedure was applied to the estimated ePDC by offsetting their value to zero at the corresponding frequency *f* if the threshold from the surrogate ePDC$\left(f,w_{l}\right)$ was higher than the estimated ePDC$\left(f,w_{l}\right)$. It is important to note that increasing the number of surrogates is a time consuming procedure when you are working with segmented multivariate process—for each subject the multivariate process was segmented in 20 time windows.

#### 2.3.3. Parametrization of Time-Frequency Representation of Interactions

For the statistical evaluation of significant group differences, several tentative parameters were extracted from the corrected ePDC function at each window $w_{l}$, as shown in Table 2. The ePDC function was parametrized in the low frequency band (LF: 0.04–0.15 Hz) regarding the interactions SYS→BBI and BBI→SYS, and in the high frequency band (HF: 0.15–0.40 Hz) considering the interactions RESP→BBI and RESP→SYS.

### 2.4. Statistics

Requirements for parametric tests, such as normal distribution or variance homogeneity, were not fulfilled in this study. Therefore, non-parametric statistical analysis via the Mann–Whitney-U-test was used to determine which of the ePDC parameters discriminated between two groups in each window (test A). Also, statistical differences between BL and OP windows were evaluated using the Wilcoxon test, followed by corrections of the *p*-values by the Bonferroni–Holm method in each group separately (test B). The most significant parameters were shown using boxplots. Boxplots represent the dynamic behavior during HUT test in terms of median, 25th percentile, 75th percentile, minimum and maximum value of the data set in each window. The statistics software was SPSS 20.0 (IBM SPSS statistics, Armonk, NY) and the significance was subdivided into three levels for descriptive purposes: slightly significant for *p* < 0.05 (*), moderately significant for *p* < 0.01 (+) and highly significant for *p* < 0.001 (#).

## 3. Results

Findings revealed high variability in the magnitude and frequency of the time-frequency representation of interactions by ePDC between subjects within each group. Therefore, in the following section, averaged time-frequency representations over all subjects within each of the four groups will be demonstrated for each interaction. The *x*-axis represents the 20 temporal windows including BL (window 1) and OP (windows 7–20). The *y*-axis refers to the frequency in Hz over the range of 0.02–0.50 Hz. The magnitude of the ePDC (*z*-axis) ranges from 0 to 0.55. Subsequently, the temporal dynamic behavior of statistically significant ePDC parameters during the HUT test will be shown for every second window to facilitate the visual interpretation. The group of male patients was excluded from statistical analysis due to their small sample size, but its tendency was incorporated in the discussion and in the corresponding figures.

### 3.1. Averaged Time-Frequency Representation by ePDC (TF-ePDC) during the HUT Test

**Figure 1:**
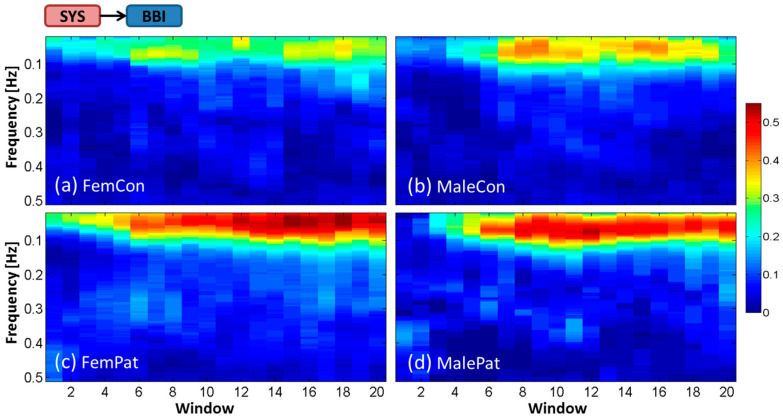
Temporal dynamic behavior of averaged ePDC for the cardiovascular interaction SYS→BBI during the head-up tilt (HUT) test for (**a**) female controls, (**b**) male controls, (**c**) female patients and (**d**) male patients.

**Figure 2:**
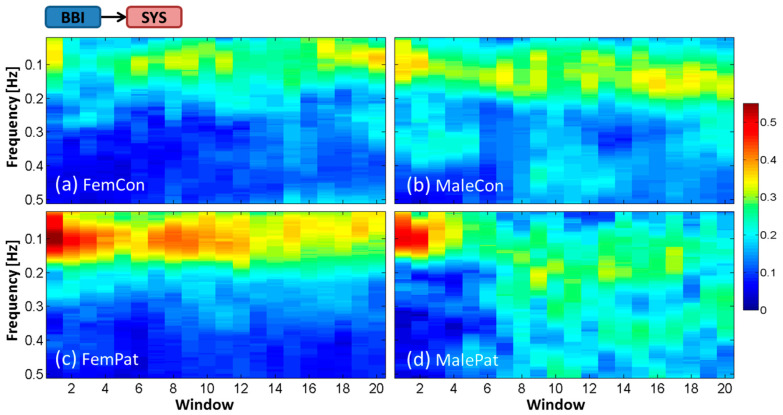
Temporal dynamic behavior of averaged ePDC for the cardiovascular interaction BBI→SYS during HUT test for (**a**) female controls, (**b**) male controls, (**c**) female patients and (**d**) male patients.

**Figure 3:**
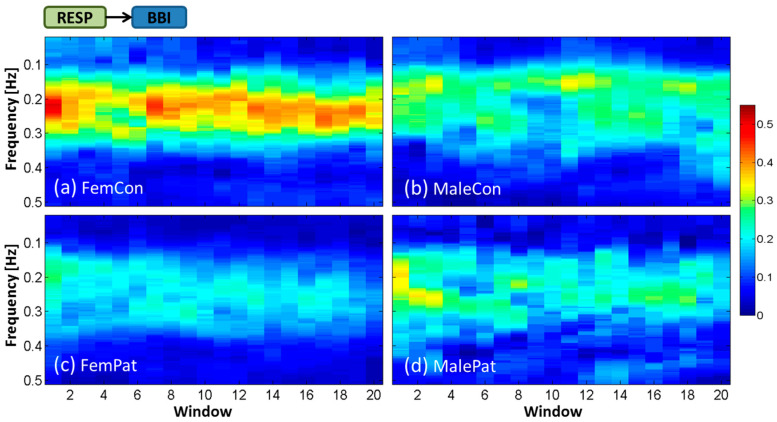
Temporal dynamic behavior of averaged ePDC for the cardiorespiratory interaction RESP→BBI during the HUT test for (**a**) female controls, (**b**) male controls, (**c**) female patients and (**d**) male patients.

**Figure 4:**
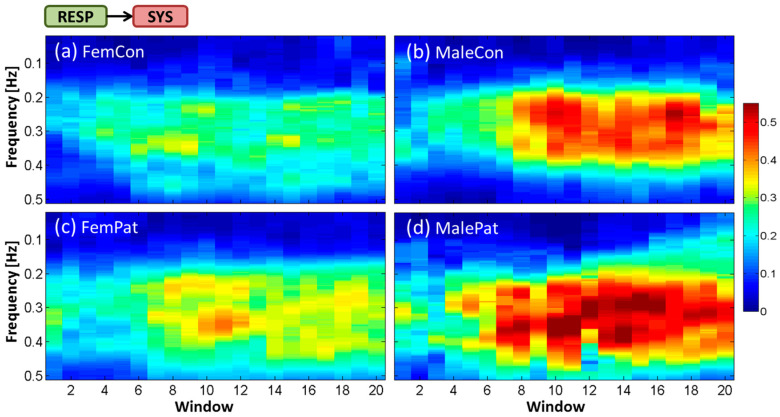
Temporal dynamic behavior of averaged ePDC for the cardiorespiratory interaction RESP→SYS during the HUT test for (**a**) female controls, (**b**) male controls, (**c**) female patients and (**d**) male patients.

The averaged TF-ePDC was obtained for the cardiovascular interactions SYS→BBI (Figure 1) and BBI→SYS (Figure 2), as well as for the cardiorespiratory interactions RESP→BBI (Figure 3) and RESP→SYS (Figure 4). The subplots refer consistently to female controls (a), male controls (b), female patients (c) and male patients (d). Regarding the interaction from SYS to BBI, the averaged TF-ePDC, labeled as ePDC_SYS__→BBI_, increased during OP compared to BL in the LF band in all four groups with a tendency of the lowest values ≈0.30–0.35 in female controls, followed by ≈0.35–0.40 in male controls, ≈0.45–0.50 in male patients and ≈0.50–0.55 in female patients during OP, as shown in Figure 1a–d. In female subjects, ePDC_SYS__→BBI_ appeared to be slightly present at ≈0.05 Hz in BL, but was absent in male subjects. During OP, the maximum peak value (MPV) of ePDC_SYS__→BBI_ was located at a frequency of ≈0.07 Hz, while 0.10 Hz constituted the upper limit of the frequency range of considerable ePDC_SYS__→BBI_ in all subjects. Male and female patients revealed the highest TF-ePDC values during OP. Male patients had their maximum at window 12, while the averaged ePDC_SYS__→BBI_ continuously increased during OP in female patients.

The averaged TF-ePDC from BBI to SYS (ePDC_BBI__→SYS_) did not change during the HUT test in controls, but was considerably increased in BL and decreased during OP in patients, as shown in Figure 2a–d. In controls, ePDC_BBI__→SYS_ showed values ≈0.30–0.35 during the complete OP, over ≈0.04–0.15 Hz in female controls and over ≈0.04–0.20 Hz in male controls. In female patients, the averaged ePDC_BBI__→SYS_ revealed values ≈0.50–0.55 around 0.10 Hz in BL, which continuously decreased during the progression of OP toward values ≈0.30–0.35 within the LF band. In male patients, mean ePDC_BBI__→SYS_ was increased as well, with ≈0.50 around 0.10 Hz, but spread over the LF and HF band during OP with values ≈0.30.

Interactions from RESP to BBI were prominent over ≈0.15–0.35 Hz within the HF band. The average value of ePDC_RESP__→BBI_ was the highest in female controls with ≈0.40–0.50, followed by male controls with ≈0.25–0.35, male patients with ≈0.20–0.30 and the lowest in female patients with ≈0.15–0.25 during the HUT test, as shown in Figure 3a–d. Controls appeared to reestablish their averaged ePDC_RESP__→BBI_, which seemed to remain decreased in patients during OP compared with BL. The differences of ePDC_RESP__→BBI_ between controls and patients were more evident in women than in men.

The interaction from RESP to SYS (ePDC_RESP__→SYS_) was pronounced during OP within the HF band; over the frequency range from ≈0.15 Hz until ≈0.40 Hz in controls and until ≈0.45 Hz in patients, as shown in Figure 4a–d. The averaged value of ePDC_RESP__→SYS_ was noticeably higher in men (≈0.40–0.55) than in women (≈0.25–0.40), with slightly higher values in patients compared with controls within the same gender groups. The highest averaged ePDC_RESP__→SYS_ values during OP were shown at windows 9 and 15 in female controls, around window 11 in female patients, at windows 10 and 17 in male controls and during windows 10–16 in male patients.

### 3.2. Dynamical Differences by TF-ePDC Parameters

Statistically significant differences were primarily obtained by the TF-ePDC parameters LF, HF, LF-max, HF-max, LF-mv and HF-mv. Gender differences in controls by test A were present during OP with regard to the cardiorespiratory interactions including RESP→BBI with an increased HF-max in female controls (windows 8–15, *p* < 0.05) as well as RESP→SYS with an increased HF-mv in male controls (windows 10–18, *p* < 0.05). The cardiovascular interactions did not reveal significant gender differences. Significant differences in women due to orthostatic intolerance were demonstrated for both cardiovascular interactions SYS→BBI and BBI→SYS as well as for the cardiorespiratory interaction RESP→BBI. Hereby, LF-max from SYS→BBI analysis was increased in female patients during OP (windows 7–13, *p* < 0.05; windows 14–20, *p* < 0.01), LF-max from BBI→SYS analysis was highly significantly increased in FemPat in BL (*p* < 0.001) and HF-max from RESP→BBI analysis was decreased in FemPat during the complete HUT test with *p* < 0.05 in BL until *p* < 0.001 during OP (window 10–13). Interaction analysis of RESP→SYS did not reveal significant differences between female groups. By trend, male patients showed an increased LF-max for the interaction SYS→BBI during some OP windows, an increased LF-max for the interaction BBI→SYS during BL, a decreased HF-max for the interaction RESP→BBI during OP and an increased HF-mv for the interaction RESP→SYS during OP in comparison to male controls.

**Figure 5:**
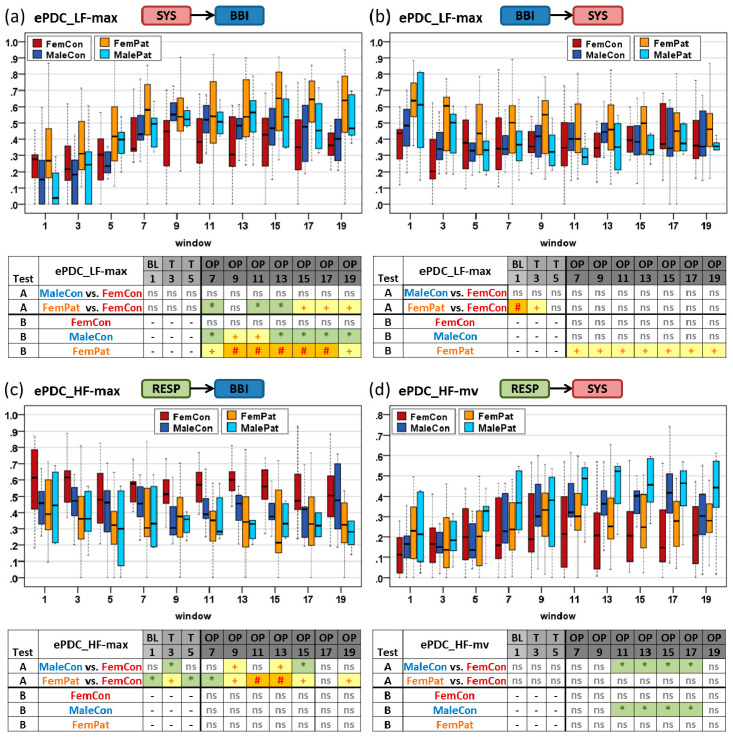
Temporal dynamic behavior of ePDC_LF-max, ePDC_HF-max, ePDC_HF-mv parameters for cardiovascular interactions SYS→BBI (**a**) and BBI→SYS (**b**) as well as cardiorespiratory interactions RESP→BBI (**c**) and RESP→SYS (**d**) during the HUT test comparing female controls, male controls, female patients and male patients. Tables in each subplot show corresponding significant differences for test A (MaleCon vs. FemCon and FemPat vs. FemCon) and test B (FemCon, MaleCon and FemPat). BL: baseline; T: transition; OP: orthostatic phase; *: *p* < 0.05; +: *p* < 0.01; #: *p* < 0.001; ns: not significant.

Significant differences by test B (BL versus OP windows) were shown in MaleCon and FemPat, but not in FemCon. The index LF-max from SYS→BBI analysis significantly increased during OP in MaleCon (windows 7–20, until *p* < 0.01) and in FemPat (windows 7–20, mainly *p* < 0.001). In addition, LF-max from BBI→SYS analysis decreased in FemPat (windows 7–20, mainly *p* < 0.01), while HF-mv from RESP→SYS analysis increased in MaleCon (windows 10–18, *p* < 0.05) during OP. In MalePat, there was a tendency of increased TF-ePDC parameters for the interactions SYS→BBI and RESP→SYS along with decreased indices for the interactions BBI→SYS and RESP→BBI during OP compared to BL. The temporal dynamic behavior of selected TF-ePDC parameters was presented in Figure 5 for every second window during the HUT test with their corresponding statistical results. The index LF-max was chosen for the interactions SYS→BBI (Figure 5a) and BBI→SYS (Figure 5b), HF-max was selected for the interaction RESP→BBI (Figure 5c) and HF-mv for the interaction RESP→SYS (Figure 5d).

## 4. Discussion

In this study, cardiovascular and cardiorespiratory interactions during the HUT test were investigated by a time-frequency representation applying the extended partial directed coherence to segmented multivariate time series consisting of heart period, systolic blood pressure and respiration. The proposed scheme aimed to find the temporal course of interactions, which reveals the adaptation of the autonomic nervous system to compensate the orthostatic stress, in other words, interactions are time-dependent, reflecting the capacity of the physiological systems to maintain adequate cardiovascular regulation and to avoid syncope. Analyses were performed to study the effect of gender as well as the effect of orthostatic intolerance in a cohort of healthy subjects and patients, both male and female. Each of the four groups presented certain intensities of direct causality, with some gender differences mainly occurring in cardiorespiratory interactions during OP, and evident differences due to orthostatic intolerance in cardiovascular and cardiorespiratory interactions during both BL and OP, especially in women.

Concerning the cardiovascular interaction SYS→BBI, results from TF-ePDC confirmed the increased information flow from SYS to BBI during OP compared with BL within LF oscillations. The increased SYS→BBI causality has been associated with increased information flow along the baroreflex and augmented sympathetic tone during orthostatic stress [3,18,22]. According to the present study, the increased SYS→BBI information flow was not significant in FemCon, slightly significant in MaleCon and highly significant in FemPat. The increased SYS→BBI flow was further present in MalePat by trend, similar to but not as high as in FemPat. These changes led to a trend of higher SYS→BBI causality in MaleCon than in FemCon during OP and, more evidently, greater SYS→BBI causality in FemPat than in FemCon. In previous studies, sympathetic activity was found to be slightly increased in MaleCon and considerably increased in FemPat during HUT test compared to FemCon [27,28]. In addition, the baroreflex function is impaired in patients with orthostatic intolerance [29]. According to these findings, results from TF-ePDC analysis during the HUT test possibly indicated that the higher the information flow from SYS to BBI, the higher the sympathetic cardiovascular modulation and the more impaired the baroreflex function becomes. Female patients were further characterized by highly significant increased blood pressure variability during OP [28], which could have exceedingly stimulated the baroreceptors and augmented the information flow from SYS to BBI. It is worthy to mention that during the analyzed 18 min of OP, patients demonstrated common symptoms of orthostatic intolerance, but not yet of emerging pre-syncope. However, causality parameters from TF-ePDC already revealed distinctive alterations in the information flow from SYS to BBI immediately after the tilt-up in female patients. Previous studies performing causality or coupling analysis determined differences in patients not before the late tilt or pre-syncopal phase [12,20,23,30]. In the present study, the application of the extended PDC including instantaneous and lagged effects, in combination with the windowing approach, as well as the separate investigation of male and female subjects, could have contributed to the early differentiation between FemCon and FemPat during the orthostatic phase of the HUT test.

The interaction BBI→SYS corresponding to the mechanical modulation has been described to be unchanged during the HUT test in healthy subjects [31]. This finding could be confirmed in male and female controls with no gender differences. However, in female patients, TF-ePDC from BBI to SYS was considerably increased already during BL in the supine position and significantly decreased during OP toward values observed in controls. A similar trend was observed in male patients. In BL, FemPat were already characterized by slightly increased sympathetic activity in comparison to FemCon. The augmented sympathetic modulation could have accounted for the increased information flow from BBI to SYS, even in the supine position in FemPat. It could be possible that previous experiences with orthostatic intolerance already modified the baseline behavior in female patients by enhancing the cardiac control on blood pressure just before imminent orthostatic stress. However, heart rate and cardiac contractility were not significantly different between FemCon and FemPat during BL. During orthostatic phase, the prior augmented cardiac control from BBI to SYS in FemPat might have been exhausted and withdrawn due to rising causality from SYS to BBI.

**Figure 6:**
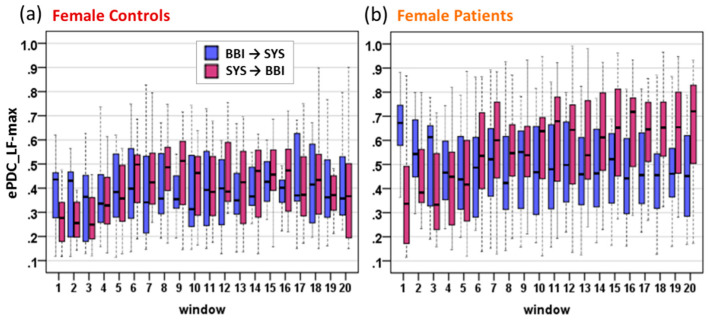
Temporal dynamic behavior of the ePDC_LF-max parameter of cardiovascular interactions BBI→SYS and SYS→BBI for female controls (**a**) and female patients (**b**) during the HUT test.

Comparing the cardiovascular causal directions in female controls (Figure 6a), the interaction BBI→SYS slightly dominated over the interaction SYS→BBI during BL, which were relatively balanced shortly after tilt-up due to the slight increase in SYS→BBI interaction during OP. In contrast, female patients (Figure 6b) revealed considerable imbalances of BBI→SYS highly dominating over SYS→BBI during BL that turned progressively into the opposite during OP with increased SYS→BBI information flow dominating over the direction BBI→SYS. It could be possible that the predominant sympathetic modulation in female patients with orthostatic intolerance generated augmented information flow in both directions in an attempt to preserve sufficiently adequate cardiovascular regulation.

Despite orthostatic intolerance being primary a cardiovascular autonomic disorder, cardiorespiratory interactions within HF oscillations were also affected in patients as well as by gender effects. The cardiorespiratory interaction from RESP to BBI representing RSA has been shown to decline during OP due to vagal withdrawal [3,18,22]. Statistically significant decreases of the TF-ePDC information flow from RESP to BBI could not be confirmed in any group in the present study. However, group differences due to gender were present in controls during OP with decreased RESP→BBI interaction in MaleCon. Moreover in FemPat, the information flow from RESP to BBI was already slightly reduced in BL and considerably during OP compared to FemCon. In men, there was only a slight tendency of decreased RESP→BBI interaction in patients during OP. Consequently, the interaction RESP→BBI, and therefore cardiac vagal modulation within HF mediated by respiration, was the most pronounced in FemCon followed by less causality in male subjects and the lowest in FemPat during the HUT test. This group order was in agreement with the averaged respiratory frequency during the HUT test at 0.26 Hz in female controls, at 0.28 Hz in male controls and at 0.30 Hz in patients. The causal relationship RESP→BBI is supposed to decrease with increased respiratory frequency due to vagal withdrawal. Based on the distinct vagal withdrawal during the HUT test, female patients were possibly not able to establish a pronounced RESP→BBI interaction useful enough to increase heart rate variability and consequently stabilize blood pressure regulation [32].

The averaged information flow from RESP to SYS within HF oscillations increased during OP compared with BL in all four groups with greater increases in men than in women. Due to high variances of the TF-ePDC parameters, slightly significant differences during OP were only obtained in MaleCon with increased RESP→SYS interaction in comparison to FemCon. Male patients demonstrated a trend of the highest RESP→SYS interaction during OP comparing all groups. The causal influence from RESP onto SYS seemed to be more affected by gender than by orthostatic intolerance. Previous studies determined unchanged RESP-SYS coupling during OP [21,33]. However, the TF-ePDC analysis in the present study indicated an increased RESP→SYS interaction after the tilt-up, more evident in men than in women. Even though significant differences between controls and patients were not obtained, male subjects might exhibit an advantage of increased blood pressure modulations mediated by respiration during orthostatic stress when cardiovascular interactions could be impaired. It is important to mention that although it is challenging nowadays to study physiological interactions, more research needs to be done to establish interactions patterns in healthy and sick populations to generate a gold standard that can be used in clinical settings.

## 5. Conclusions

In this study, the extended partial directed coherence has been shown to be a useful linear technique to analyze the temporal dynamic behavior of cardiovascular and cardiorespiratory interactions during orthostatic stress. Gender differences in healthy subjects were present in cardiorespiratory interactions during the orthostatic phase without compromised autonomic regulation. However in patients, analysis by TF-ePDC revealed considerable dynamic alterations mainly within cardiovascular interactions, but in cardiorespiratory interactions as well, over the temporal course of a head-up tilt test. Considering young female patients with orthostatic intolerance, the information flow from heart rate to systolic blood pressure (mechanical modulation) was already increased before the tilt-up, the information flow from systolic blood pressure to heart rate (neural baroreflex) increased during the orthostatic phase, while the information flow from respiration to heart rate (respiratory sinus arrhythmia) was decreased during the complete head-up tilt test. The last two causality directions may include instantaneous effects in addition to lagged effects, which highly endorsed the use of the extended PDC. It is plausible that the information provided by the estimation of the dynamics of the cardiorespiratory and cardiovascular interactions obtained by TF-ePDC may be used clinically as the physicians currently employ the analysis of the heart rate variability, but more research needs to be done to validate these concepts and to generate a gold standard. Finally, future studies should account for time-variant extended models that could be applied to nonlinear and non-stationary data.

## Figures and Tables

**Table 1:** Age range of included study groups.

Group	Name	Number of Subjects	Age (years)	Assigned Color
Female Controls	FemCon	13	26 ± 5	Red
Male Controls	MaleCon	13	28 ± 3	Blue
Female Patients	FemPat	21	28 ± 7	Orange
Male Patients	MalePat	6	26 ± 6	Light blue

**Table 2:** Extracted parameters from extended partial directed coherence (ePDC) analysis.

Parameter	Low Frequency Band SYS→BBI, BBI→SYS	High Frequency Band RESP→BBI, RESP→SYS
Percentile frequencies 25%, …, 95%	ePDC_LF-pf25, …, ePDC_LF-pf95	ePDC_HF-pf25, …, ePDC_HF-pf95
Absolute power	ePDC_LF	ePDC_HF
Maximum peak value	ePDC_LF-max	ePDC_HF-max
Frequency of MPV	ePDC_LF-fmax	ePDC_HF-fmax
Mean value	ePDC_LF-mv	ePDC_HF-mv

SYS: systolic blood pressure; BBI: beat-to-beat intervals; RESP: respiratory amplitude at BBI onset.
